# Design, Development and Application of a Modular Electromagnetic Induction (EMI) Sensor for Near-Surface Geophysical Surveys

**DOI:** 10.3390/s24134159

**Published:** 2024-06-26

**Authors:** Luzian Wolf, Adrian Flores Orozco

**Affiliations:** Research Unit Geophysics, Department of Geodesy and Geoinformation, TU Wien, 1040 Vienna, Austria; adrian.flores-orozco@geo.tuwien.ac.at

**Keywords:** electromagnetic induction, low induction number, EMI, terrain conductivity meter, geophysics, near-surface survey, agrogeophysics, hydrogeophysics, open hardware

## Abstract

Low-frequency electromagnetic induction (EMI) is a non-invasive geophysical method that is based on the induction of electromagnetic (EM) waves into the subsurface to quantify changes in electrical conductivity. In this study, we present an open (design details and software are accessible) and modular system for the collection of EMI data. The instrument proposed allows for the separations between the transmitter to be adjusted and up to four receiving antennas as well as the acquisition frequency (in the range between 3 and 50 kHz) to permit measurements with variable depth of investigation. The sensor provides access to raw data and the software described in this study allows control of the signal processing chain. The design specifications permit apparent conductivity measurements in the range of between 1 mS/m and 1000 mS/m, with a resolution of 1.0 mS/m and with a sampling rate of up to 10 samples per second. The sensor allows for a synchronous acquisition of a time stamp and a location stamp for each data sample. The sensor has a mass of less than 5 kg, is portable and suitable for one-person operation, provides 4 h of operation time on one battery charge, and provides sufficient rigidity for practical field operations.

## 1. Introduction

The electromagnetic method at low induction number was initially proposed by [1] as a non-invasive geophysical method that can resolve for the electrical conductivity (σ) of the near surface, based on measurements of the response of the subsurface to a low frequency electromagnetic field. Commonly referred to as terrain conductivity-meters [1], or EMI [2], the method has been used for over four decades for soil investigations (see, e.g., [3], and references therein). Recent investigations exploit the use of relatively small instruments with a number of measuring coils in different orientation [4] to collect data with high resolution and varying depth of investigation, making the method attractive for agricultural and hydrogeological studies (e.g., [5,6,7,8]). Other applications of the method range from the investigations of landslides [9], landfills [10], archeological sites [11,12,13] and the investigation of contaminant plumes [14]. A few studies have also used the method for monitoring purposes [15,16,17].

Most of the applications reported have employed commercial devices, which feature good accuracy and provide a trade-off between robustness and production costs. Available EMI instruments in the market often also provide interfaces for the collection and processing—including inversion—of the data. However, commercial instruments are usually designed with a fixed separation between transmitter and receiver antennas, and investigations for variable depths of investigations require the purchase of multiple devices. Moreover, commercial instruments usually do not (to the best of our knowledge) provide access to important device operating parameters, to raw data or to the signal processing chain.

The company GF Instruments (Brno, Czech Republic) has developed the family of portable single operator CMD-Explorer systems [18] with a depth range of between 0.15 m and 6.7 m. The CMD-Mini Explorer [19] is a compact instrument with a length of 1.3 m, a diameter of 5 cm, a working weight of 2 kg, and is operating at a frequency of 30 kHz. The instrument is equipped with 3 receiver antennas, arranged at a distance of 0.32 m, 0.71 m, and 1.18 m from the transmitter antenna. Alternatively, the CMD-Explorer has a length of 4.85 m, a diameter of 10 cm, a working weight of 8 kg, and is operating at a frequency of 10 kHz. The instrument is equipped with three receiver antennas, arranged in a distance of 1.48 m, 2.82 m, and 4.49 m from the transmitter antenna. The two-operator system CMD-DUO uses a separate transmitter and receiver coil antenna with a diameter of 65 cm, a working weight of 9.5 kg for the transmitter, and 6.2 kg for the receiver. Antenna coils are linked with a cable that can be adjusted in length for antenna distances of 10 m, 20 m, and 40 m.

The company Geonics Ltd. (Mississauga, ON, Canada) supplies comparable instruments with the systems EM31, EM34, and EM38 [20]. The company Dualem Inc. (Milton, ON, Canada) supplies several types of portable systems with transmitter–receiver coil separations of between 1 and 8 m. These systems often include an auxiliary sensor that record environmental variables such as instrument position and orientation, temperature, and the ambient magnetic field. GSSI Geophysical Survey Systems (Nashua, NH, USA) supplies the Profiler EMP-400 instrument, with a coil separation of 1.2 m, and is equipped to perform EMI measurements at up to three frequencies simultaneously.

In addition, experimental systems have been built and tested. A frequency-domain EMI system was built at the Department of Geophysics, Colorado School of Mines, Golden, CO, USA [21]. According to the authors, the proof-of-concept instrument has material costs of under US$400 and is capable of sensing conductive objects in near-surface environments. The functionality of the system was tested in a controlled laboratory setting and validated over a conductive target in an outdoor environment, at frequencies of 0.4 kHz and 1.6 kHz, and transmitter–receiver antenna distances of up to 25 m.

A modular EMI system was developed by the Central Institute for Electronic Systems, Forschungszentrum Jülich GmbH, Jülich, Germany [22,23]. This system is designed to measure the very small secondary magnetic field without analogue compensation of the primary signal, thereby enabling flexible sensor configurations with quantitatively comparable measurement data, and is optimized for flexible setups with separations between transmitter and receiver coils of 1.0 m. In addition, a model-based drift correction method that uses measured electrical system parameters was developed and tested. Error sources caused by environmental and instrumental effects were analyzed and a method to select optimal measurement frequencies based on an ambient noise level analysis was developed.

In this work, we describe the design, implementation and application of an experimental Modular Electromagnetic Induction System (MEMIS). Many components and sub-systems used in this development are commercially available off-the-shelf items. The design of the proposed instruments aims at becoming a low-cost alternative for research and academia, where access to a large pool of commercial instruments might be limited. As such, the proposed MEMIS is not intended to compete with commercial devices, as the production costs, field robustness and libraries for the processing of the data might increase its production costs. Nevertheless, the proposed instrument might help to enhance the resolution of the EMI method for shallow investigations, as the geometry of the device can be adjusted to enhance the sensitivity in the shallow soil horizons.

The MEMIS, as presented here, supports up to four receiver coil antennas for the simultaneous recording of (apparent) conductivity ($\sigma_{a}$) at four different depths, which here have been tested up to 6.0 m. Position and orientation are measured with an integrated GPS module/inertial motion unit (IMU). Signals from the antennas and auxiliary sensors are recorded without pre-processing in analog format with a bandwidth of 50 kHz. The interpretation of GPS and IMU data sentences, filtering of antenna signals, calculation of signal attenuations, phase differences, and apparent conductivities are performed during post-processing in the digital domain with a dedicated software program. With a mass of less than 5 kg, the device is easy to handle, is suitable for one-person operation, and its build is sufficiently rugged for practical field operations.

## 2. Materials and Methods

### 2.1. The Low Induction Number Electromagnetic Method

Electromagnetic induction at the low induction number (EMI-LIN) is a non-invasive geophysical method that resolves the (bulk) electrical conductivity $\sigma_{a}$ of a certain volume of the subsurface. The method was initially presented by McNeill (1980) [1]. It is based on the circulation of an alternating current in a transmitter coil at a given frequency (f), given by the following:

(1) $$I\left(t\right)=I_{0}sin(\omega t)$$

In Equation (1), ω represents the angular frequency corresponding to the excitation frequency (f) of the current circulating in the transmitter (ω=2πf), t represents the time (in seconds) and $I_{0}$ is the amplitude of the current injected. This current induces a sinusoidal (harmonic) magnetic field B(t,x,y,z). Temporal changes of this “primary” magnetic field (dB/dt) interact with the electrical charges in the subsurface, inducing eddy currents, which are proportional to the temporal variation of the primary magnetic field *d**B***/*dt* and the electrical conductivity *σ* of the subsurface material [24]. Eddy currents induce a secondary magnetic field that has a phase delay of $\frac{\pi }{2}$ relative to the primary field and superimposes to it. This interaction causes (1) attenuation and (2) a small phase delay of the magnetic field.

The magnetic field resulting from the superposition of the primary and secondary field is sensed by a receiver loop antenna, which has a distance *s* to the transmitter. The receiver coil yields an open-loop voltage $U\left(t\right)$ given by the following:

(2) $$U\left(t\right)=U_{0}sin(\omega t+\frac{\pi }{2}+\Delta \varphi )$$

The phase delay of 90° (π/2) in Equation (2) originates from the link between electric (E) and magnetic (B) fields described by Faraday’s law $\left(rot\left(E\right)=-dB/dt\right).$ The phase delay ∆φ of the magnetic field between transmitter and receiver contains information about the electrical conductivity of the subsurface materials (${∆\varphi }_{MAT}$). However, other components contribute to ∆φ as well. An additional phase delay ${∆\varphi }_{SYS}$ arises from delays in the electronic components of the receiver signal processing chain, and from the finite propagation time of the EM wave between the transmitter and the receiver. Hence, ∆φ can be described by the following:

(3) $$∆\varphi ={∆\varphi }_{SYS}+{∆\varphi }_{MAT}$$

${∆\varphi }_{SYS}$ has a specific value for each receiver antenna and for each operating frequency of the sensor and can be determined through a calibration procedure.

Electromagnetic waves attenuate when propagating in conductive media [25]. This attenuation is measured by the so-called skin depth (δ), which is defined as the distance at which the amplitude of the incident EM wave has decayed to 1/e (ca~37% of the initial amplitude) [25]. The skin depth is a function of the electrical conductivity (σ) and magnetic susceptibility (μ) [25,26]. Due to the low magnetic properties of common soils and geological media, most of the geophysics literature assumes the magnetic susceptibility of vacuum ($\mu_{0}$) [1,27]; thus, permitting the equation for the skin depth [26] as follows:

(4) δ=2μ0·σ·ω

The ratio between transmitter–receiver separation s and the skin depth *d* is called the induction number *b*. It is a dimensionless number, given by the following:

(5) b=sδ=s∗μ0·σ·ω2

EMI instruments working at the so-called low induction number are designed to warrant that b << 1 [1]. For such systems, the apparent electrical conductivity can be computed from the phase delay ${∆\varphi }_{MAT}$ [1]. The so-called apparent conductivity ($\sigma_{a}$) represents a weighted average value of the one-dimensional vertical conductivity distribution of the subsurface, assuming a homogeneous medium:

(6) $$\sigma_{a}=\frac{4}{\mu_{0}\cdot \omega \cdot s^{2}}\;\cdot \;\frac{H_{S}}{H_{P}}=\frac{4}{\mu_{0}\cdot \omega \cdot s^{2}}\;\cdot \tan\left({∆\varphi }_{MAT}\right)$$

Portable rigid beam EMI systems commonly use two coil arrangements [28]: (1) the horizontal co-planar coil arrangement (HCP), where both transmitter and receiver coil are oriented horizontally to the surface, and (2) the vertical co-planar coil arrangement (VCP), where coils are oriented vertically to the surface. The depth of exploration *d* is the depth at which a defined percentage of the measured signal can be attributed to the material located between the surface and depth *d* [28]. If this percentage is chosen as 70%, then the depth is *d =* 1.6 *s* for the HCP arrangement, and *d* = 0.76 *s* for the VCP arrangement [29,30].

### 2.2. MEMIS—Functional Design

**Figure 1:**
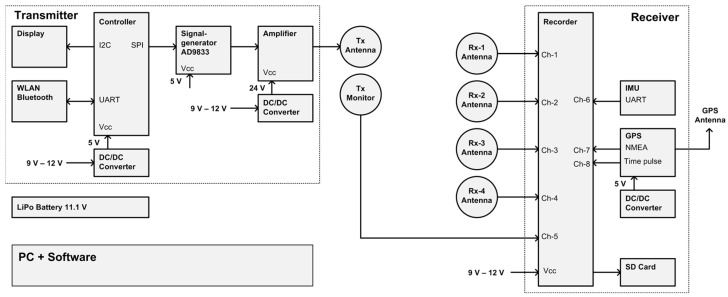
Hardware design of the Modular EMI System: The sensor comprises a variety of components that can be grouped in three sections: (1) the transmitter (**left-side**), (2) the receiver (**right-side**), and (3) software for processing and managing of the recorded data (**bottom left**). The label ‘V_CC_’ indicates the positive supply voltage input of sub-systems, ‘UART’ (Universal Asynchronous Receiver/Transmitter) indicates an asynchronous bi-directional serial communication port, ‘I2C’ (Inter-Integrated Circuit) indicates a standardized two-wire communication port between electronic components, ‘SPI’ (Serial Peripheral Interface) indicates a three-wire synchronous serial communication port between electronic components, and ‘WLAN’ (Wireless Local Area Network) is a network connection for system extensions.

The design of the proposed MEMIS comprises of three sections: a transmitter, a receiver and software for the management of the data and conversion into $\sigma_{a}$. Each section groups different components as illustrated in Figure 1.

The transmitter consists of a signal generator, an amplifier, a microcontroller unit, a small display, a WLAN and Bluetooth interface for optional future extensions, power conditioning electronics, and a coil antenna (Tx). The signal generator and amplifier produce a sinusoidal signal that is conducted to the transmitter antenna (Tx). The signal generator itself is controlled by a microcontroller, to which a rudimentary user interface—consisting of a small display and a push button—is connected. With this interface, the user can cycle through a list of pre-defined frequencies by pressing a push button. Below, we provide further details on the different components.

The receiver section consists of an analog multi-channel data recorder, a transmitter monitoring coil antenna (Tx-Mon), up to four receiver coil antennas (Rx-1 to Rx-4), power conditioning electronics, a GPS unit with antenna to provide information about the geo-position of the instrument, and an inertial motion unit (IMU) to measure the orientation of the instrument. Signals sensed by the receiver coil antennas (Tx-Mon, Rx-1 to Rx-4) are recorded without pre-processing with the analog recorder. The binary signals originating from the GPS unit and IMU are recorded without pre-processing with the analog recorder as well. Instead of measuring the transmitter current directly, the receiver contains a monitoring antenna (Tx-Mon) that is placed close to the transmitting coil antenna. This approach provides galvanic isolation between the transmitter and receiver section, which simplifies the electronic design by decoupling signal offset levels and increases the flexibility to use different transmitter amplifiers. All data are recorded to the SD card of the recorder. The analysis of recorded data is performed after a field measurement has been completed with dedicated post processing software (for details see Section 2.3). Transmitter and receiver are powered by a rechargeable 12 V battery.

The MEMIS features a broad-band design. Signals from the antennas, the GPS unit, and the IMU are recorded without pre-processing with a bandwidth of 50 kHz. The parsing and interpretation of GPS and IMU data sentences, filtering of antenna signals, calculation of signal attenuations, phase differences and apparent conductivities are performed during post-processing in the digital domain with a dedicated software program. This approach keeps the hardware design simple, as it relies—to a large extent—on commercially readily available components and sub-systems, while more complex processing steps are implemented in software.

### 2.3. MEMIS—Implementation

**Figure 2:**
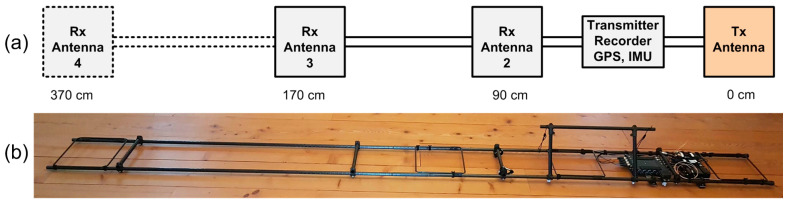
The proposed MEMIS: (**a**) schematic diagram explaining the main components and the dimensions; (**b**) picture of the device.

The MEMIS presented here comprises one transmitter and three receiver antennas with transmitter-receiver coil separations of 90 cm, 170 cm and 370 cm, as presented in Figure 2. The device can be extended to increase or decrease the separation between transmitter and receiver antennas, or to add additional receiver antennas.

The signal generator is implemented with the electronic component AD9833 from Analog Devices Inc. (Wilmington, MA, USA) [31], a low-power, programmable waveform generator. The device is capable of producing sine, square or triangular waves with frequencies from DC to 12.5 MHz. The device’s frequency register is 28 bits wide, resulting in a frequency resolution higher than 0.1 Hz over the whole frequency range. An external 12.5 MHz quartz crystal defines the frequency reference of the device. The actual output frequency and waveform type (sine, triangle, or rectangular) of the device are set by writing suitable values to the 28 bits wide frequency register and the 16-bit wide control register. This task is implemented with a microcontroller type ESP32, which communicates with the waveform generator via a three-wire SPI bus (Serial Peripheral Interface). A rudimentary user interface, consisting of a small display and a push button, connects to the microcontroller. The user can cycle through a list of pre-defined frequencies between 3 kHz and 30 kHz by pressing the push button.

The amplifier is implemented with a 50 W digital amplifier module that integrates the TPA3116D2 digital amplifier chip from Texas Instruments Inc. (Dallas, TX, USA) [32]. This amplifier first converts the analog input signal into a pulse width modulated signal with a modulation frequency of approximately 500 kHz, digitally amplifies the modulated signal by rapidly switching the power transistors in the output stage back and forth between the supply rails and passes this digitally amplified signal through a low-pass filter to create the analog output signal. This approach achieves high power efficiency together with low heat dissipation because power transistors are always either fully on or fully off. The amplifier module input is directly connected to the output of the signal generator. The amplifier module is supplied with a voltage of 20 V DC which is provided by the battery via a DC/DC converter. At a frequency of 10 kHz, the amplifier can feed a maximum current of approximately 1000 mA through the transmitter coil antenna.

At operating frequencies of between 3 kHz and 30 kHz, transmitter–receiver antenna coil distances s of between 50 cm and 500 cm, and a relative magnetic susceptibility of the surrounding medium close to 1, the system is operating in the reactive near field (s << wavelength) [25]. In this region, the magnetic flux density (magnetic field) decreases rapidly with close to the 3rd power of the distance s [25].

The recorder is implemented with a professional eight-channel audio field recorder from the company Sound Devices, LLC (Reedsburg, WI, USA) [33]. This recorder provides all the functionality required to digitize and record the antenna signals. In particular, it features eight balanced analog inputs with low noise preamplifiers, adjustable gain control for each input channel, 32-bit analog to digital conversion, a maximum sample rate of 192 kSamples/s, recording to an SD card, a color display and a joystick for convenient recorder control, and plenty of other useful features. Color LEDs provide visual feedback of the signal amplitudes on each input channel.

The GPS unit is implemented with the dual band GPS receiver module ZED-F9P from uBlox (Thalwil, canton of Zürich, Switzerland) [34]. This module outputs position and time information in the form of NMEA messages (National Marine Electronics Association), a quasi-standard in GPS receivers [35,36]. The receiver has been configured to output messages of type NMEA-GGA (GPS fix data) and NMEA-RMC (recommended minimum content), which contain position and time information (latitude, longitude, elevation, UTC time), as well as quality information (the number of satellites and the technique being used by the GPS receiver to determine its location, and the age of correction information if available and used). This signal is generated with at a bitrate of 9600 bits per second, and a data rate of two messages per second. In addition, the GPS module generates a time pulse signal and supplies it at a dedicated output pin. This time pulse is synchronized to GPS time with high accuracy and has been configured to send an output signal once per second. NMEA data output and the 1 s time pulse output are connected directly to recorder inputs. At a bitrate of 9600 bits per second, each bit of an NMEA message will be sampled 20 times (192 kSamples/s/9600 bits/s = 20 Samples/bit) by the recorder digitizer. Decoding of recorded NMEA messages and the extraction of position and time information are performed after a measurement with dedicated software, as described in the Section 2.3. An active dual band GPS patch antenna is attached in the central position to a circular ground plane disk with a diameter of 10 cm and mounted to the support structure. The selected GPS unit is capable of performing Real Time Kinematics (GPS-RTK) to increase the positioning accuracy relative to a base station. An 865 MHz receiver is connected to the GPS receiver to receive RTCM (Real Time Correction Message) signals that are transmitted from a base station. The horizontal positioning accuracy of the GPS receiver depends on a variety of factors such as satellite geometry and visibility (objects in the line of sight between satellite and receiver will block satellite signals), atmospheric conditions, and whether augmentation systems are used or not. Without the utilization of RTCM signals from a base station, the average horizontal positioning accuracy can be expected to be 1.5 m [34], while with the utilization of RTCM signals, the average horizontal positioning accuracy can reach 10 cm (relative to the base station) [34].

The Inertial Motion Unit (IMU) measures the orientation of the instrument in 3D space. It is implemented with the orientation sensor WT901-RS232 from the company Wit-Motion Shenzhen Co., Ltd. (Shenzhen, China) [37]. This module integrates a three-axis gyroscope, a three-axis accelerometer, a three-axis geomagnetic sensor, and a microprocessor that fuses sensor data using a Kalman filter algorithm. This sensor has been configured to calculate and output rotation angles around the x-, y-, and *z*-axis, with a bitrate of 9600 bits per second, and a data rate of 10 messages per second. The data output of the IMU is connected directly to a recorder input, and like for the NMEA signal, extraction of rotation angle information is performed after a measurement with dedicated software (see Section 2.3).

**Figure 3:**
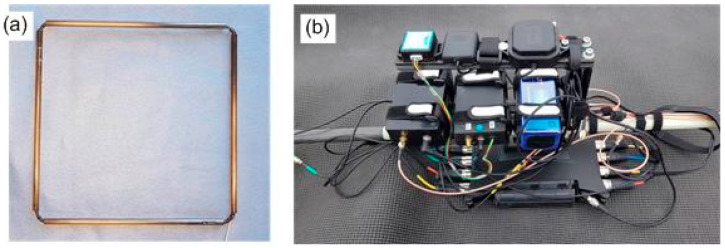
Pictures of the electronic sub-system: (**a**) receiver coil antenna; (**b**) assembled signal generator, amplifier, recorder, GPS unit, IMU, and battery.

Coil antennas for the transmitter and receiver (Figure 3a) are built with glass fiber tubes with a length of 25 cm and an inner diameter of 6 mm. The tubes are arranged in a rectangle (Figure 3a), and between 1 and 20 turns of insulated copper wire with a diameter of between 0.5 and 0.9 mm are fitted through the tubes to form the antenna loop (see Table 1 for design details). A two-component epoxy-resin is used to bond copper wires and tubes together. Each antenna coil is connected to a RG174 coaxial cable, which is terminated on its free end with a male SMA screw connector. The transmitter antenna is fitted with an auxiliary monitoring coil, in a co-axial position to the Tx antenna coil, to monitor the magnetic field generated by the transmitter antenna. The output signals of all receiver antennas and the Tx monitoring coil are in-phase to each other. Antenna outputs are connected directly to a recorder input. The rectangular design of the antennas, with copper wires enclosed in protective tubes, provides ruggedness, and facilitates the mount of antennas to the mechanical support structure.

The instrument is powered from a rechargeable 11.1 V, 5000 mAh lithium polymer battery, which is equipped with a female XT60 connector. Several DC/DC converters are used to transform the battery voltage to the required supply voltage levels of sub-systems.

A light-weight mechanical support structure complements the system. This support structure consists of a pair of glass fiber tubes with an outer diameter of 20 mm and a wall thickness of 1 mm. Tubes are arranged in parallel in a distance of 28 cm from each other and joined with T-mounts. A pair of 200 cm long tubes are used for the instrument’s standard configuration, which can be extended through extra tubes to increase the separation between the transmitter and receivers. The antennas are fixed to the support structure with adhesive tape. The assembly of electronic sub-systems is mounted to the tubes with clamps, and loose wires are fixed with Velcro straps. Receiver antennas can be mounted in at a distance of between 50 cm and 370 cm from the transmitter antenna (see Table 1 for the default configuration). Electronic subsystems (signal generator, amplifier, recorder, GPS unit, IMU, and battery) are arranged together on a metallic rail and mounted to the support structure as a single unit as depicted in Figure 3b. The system is powered up by mating the female battery connector with its male counterpart which is connected to the electronic assembly. Data recording is started and stopped with a joystick on the recorder.

### 2.4. MEMIS—Data Processing

**Figure 4:**
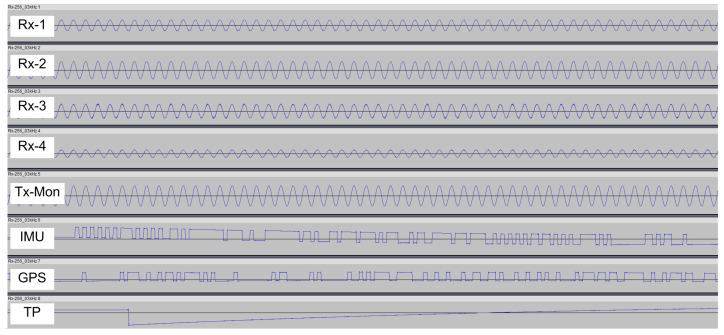
Exemplary signal records, corresponding to 25 milliseconds of data in an eight-channel wav. file. Traces Rx-1 to Rx-4 show the analog signals originating from the receiver antennas, Tx-Mon the analog signal from the transmitter monitor, IMU the data stream from the inertial motion unit, and GPS and TP the digital NMEA data stream and the time pulse signal from the GPS unit. The amplitudes of the signals are determined during post-processing by combining recorded values with recorder gain and calibration parameters.

During a measurement, the data recorder stores data streams originating from up to five coil antennas (Tx-Mon, Rx-1, Rx-2, Rx-3, Rx-4), the GPS receiver (NMEA messages, time pulse), and the inertial motion unit (IMU) in analog format in eight-channel .wav files (illustrated in Figure 4). Recording is performed with a default sampling rate of 192 kSamples/s and a bit-depth of 32 bit. At these settings, one minute of recorded raw data occupies 300 Mbytes of storage. The upper size limit of the file system used by the recorder is 4 GByte and limits the recording time to 14 min. The .wav file format consists of a header block and a data block. The format is well documented [38] and writers and readers of .wav files are relatively easy to implement in software.

Recorded data are processed with a dedicated software package that has been developed for the MEMIS. This software implements all required processing steps to transform raw data to a data table that contains apparent conductivities ($\sigma_{a}$) together with time, position, and orientation tags:

Step 1: The .wav file header is read and interpreted to obtain context information that is required to process the data stream, namely: the data format, the number of channels, the sampling rate, and the data size and duration of the recording. These values are used to set up data buffers that are required in the following processing steps.

**Figure 5:**
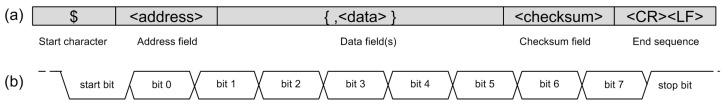
Exemplary format of the NMEA message sent by the GPS unit: (**a**) standard NMEA message; (**b**) framing of 1 byte.

Step 2: The NMEA signal track is read and analyzed. NMEA messages sent by the GPS receiver are based on NMEA 0183 Version 4.10 standard [39] An overview of this protocol is provided in [35,36]. The NMEA messages used in this application consist of approximately 80 bytes that are output from the GPS receiver module via a serial port. The first byte of a message is a ‘$’ character (Figure 5). This start character is followed by two characters that indicate the message source, or talker ID (e.g., ‘GP’ for GPS data), then followed by characters that indicate the message content (e.g., ‘RMC’ for ‘Recommended Minimum Content’), then followed by message-specific data fields. A message is terminated with the character ‘*’, followed by a one-byte hexadecimal check sum, followed by the characters <CR> (carriage return) and <LF> (line feed). Each single byte starts with one start bit, which is followed by eight data bits, and ends with one stop bit (Figure 5). At a bitrate of 9600 bits per second and a sample rate of the recorder of 192 kSamples/s, each bit of an NMEA message is represented by 20 samples (192 kSamples/s/9600 bits/s = 20 samples/bit) in the recorded data stream.

The NMEA data stream is correlated with a positive step function to detect the leading edge of start bits. The eight data bits that follow a start bit are then detected and joined to a data byte, and consecutive data bytes are joined to an NMEA message candidate. Each message candidate is tested for validity by testing for the presence of the start character ‘$’ and the end sequence <CR><LF>, a valid address field, and a valid check sum. If the validity check succeeds, the data fields of the message are interpreted and the variables date, UTC time, latitude and longitude are extracted.

Step 3: In a similar way, the time pulse signal is detected by digitally correlating the signal with a unit step function. The transition of a time pulse marks the start of a second in UTC time (coordinated universal time). This is important for accurate timing, because the start of an NMEA message typically has some delay to the start of a second in UTC time.

Step 4: The IMU signal track is read and analyzed. Messages originating from the IMU sensor have a constant length of 44 bytes, contain information about rotation angles, and follow a proprietary protocol [37]. Each single byte starts with one start bit, which is followed by eight data bits, and ends with one stop bit. At a bitrate of 9600 bits per second and a sample rate of the recorder of 192 kSamples/s, each bit of an IMU message is represented by 20 samples (192 kSamples/s/9600 bits/s = 20 samples/bit) in the recorded data stream. The IMU data stream is correlated with a positive step function to detect the leading edge of start bits. The eight data bits that follow a start bit are then detected and joined to a data byte, and consecutive data bytes are joined to an IMU message. The data fields of an IMU message are interpreted and three rotation angle variables are extracted.

Step 5: The antenna signal tracks (Tx-Mon, Rx1–Rx4) are read. Each data sample receives a time, position and an orientation stamp. Antenna data are saved in an intermediary binary file. For processing convenience, this file is split into sub-files with a typical duration of 1 min each. Sub-files have a size of 900 Mbyte per minute of raw data.

**Figure 6:**
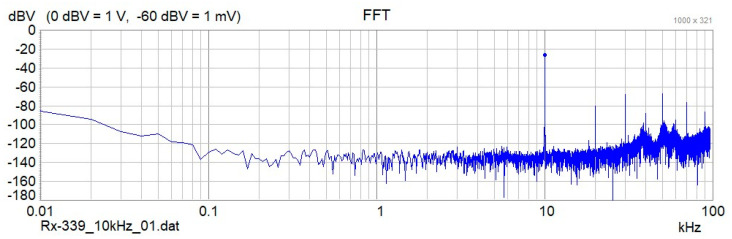
Amplitude values of the Fast Fourier Transform (FFT) of a data segment containing 100 ms of data. The plot shows a distinct peak at 10 kHz, which corresponds to the frequency of the primary magnetic field.

Step 6: A frequency analysis of the recorded antenna signals is performed. Data are read from the intermediary binary files and split into segments of 100 ms each, which corresponds to 19,200 data records. Data segments of each antenna signal (Tx-Mon, Rx1–Rx4) are first multiplied with a Hanning Window, to limit spectral leakage that is caused by calculating the FFT of a finite data sample [40]. Then the complex Fast Fourier Transform (FFT) of each data segment is calculated (Figure 6), and FFT amplitude and phase at the operating frequency are extracted from the calculated FFT arrays.

Step 7: Phase differences between the receiver signals and Tx signal are calculated, resulting in phase difference value ∆φ(k) for each Rx channel and for each 100 ms time increment k. Phase differences $∆\varphi \left(k\right)$ for each receiver are corrected with calibration data, yielding phase delays $∆\varphi_{Mat}\left(k\right)$ that are attributed to subsurface conductivities.

Step 8: Apparent electrical conductivity values are calculated for each phase difference $∆\varphi_{Mat}$, i.e., for each receiver, with Equation (6).

Step 9: Time, position, orientation, signal amplitude, signal phase difference, and conductivity values are arranged into data records for each 100 ms raw data segment and exported to a .csv text file.

## 3. Results: Field Survey with the MEMIS and Comparison with Commercial Instruments

### 3.1. Example 1: Measurement of a 400 m Long Line

To illustrate the functionality of the MEMIS, we present here the data measured along a line with a length of 400 m at a lake access road located in the national park Neusiedlersee—Seewinkel in the eastern part of Austria. At a distance of 70 m from the starting point of the line, there is a wooden bridge with a length of 7 m that leads over a shallow creek. The last 30 m section of the line corresponds to a wooden landing stage that is located above the water of the lake.

**Figure 7:**
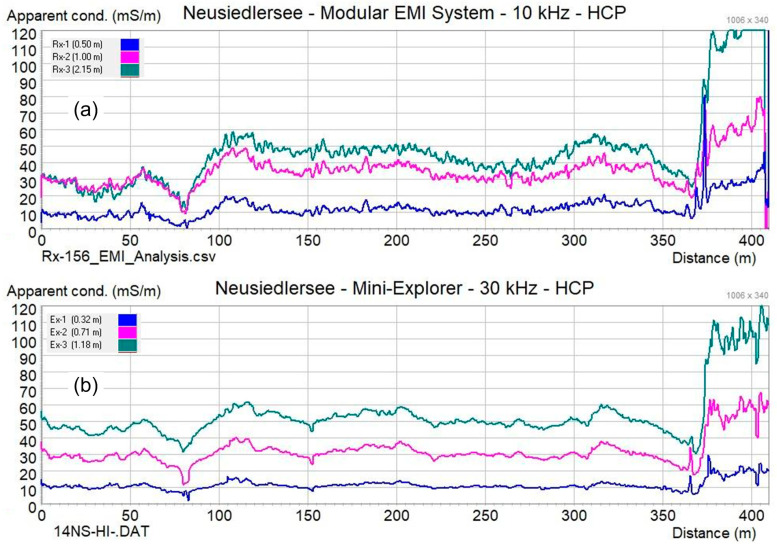
Apparent electrical conductivity (mS/m) measured along a 400 m long line in the shore of the lake Neusiedlersee. Measurements were performed with (**a**) the proposed MEMIS and (**b**) the CMD-Mini-Explorer from GF-instruments.

The line was measured with the MEMIS with a frequency of 10 kHz and transmitter-receiver coil separations of 50 cm, 100 cm, 215 cm, and 370 cm. Further measurements were conducted with the CMD-Mini-Explorer (manufactured by GF-instruments) to evaluate the quality of the data collected with the proposed instrument. The CMD-Mini-Explorer works with a frequency of 30 kHz and transmitter–receiver coil separations of 32 cm, 71 cm, and 118 cm. Measurement results are presented in Figure 7, showing apparent electrical conductivity (mS/m) plotted over distance. The visual comparison of conductivity plots from both systems shows consistency in the readings, although the operating parameters of compared instruments are somewhat different. Anomalies caused by the bridge and the landing stage are clearly visible in all measurements. The data that were used to create Figure 7 are provides as supplementary material (see section ‘Supplementary Materials’).

### 3.2. Example 2: Mapping of a 25 m × 25 m Experimental Area

**Figure 8:**
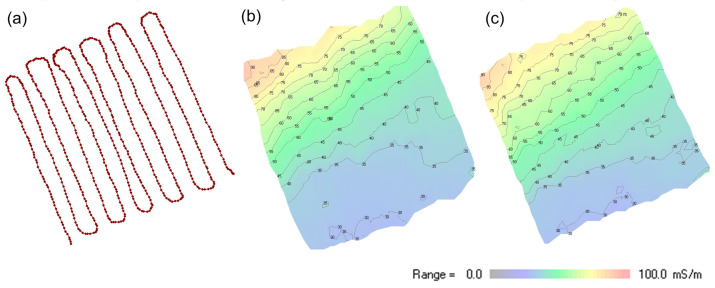
Apparent electrical conductivity (mS/m) of a 25 m × 25 m mapping area located on the shoreline of the ‘Apetloner Meierhoflacke’. The measurement locations are presented to the left (**a**), with the $\sigma_{a}$ obtained with the (**b**) MEMIS (coil separation 170 cm) and (**c**), the CMD Explorer (coil separation 148 cm).

**Figure 9:**
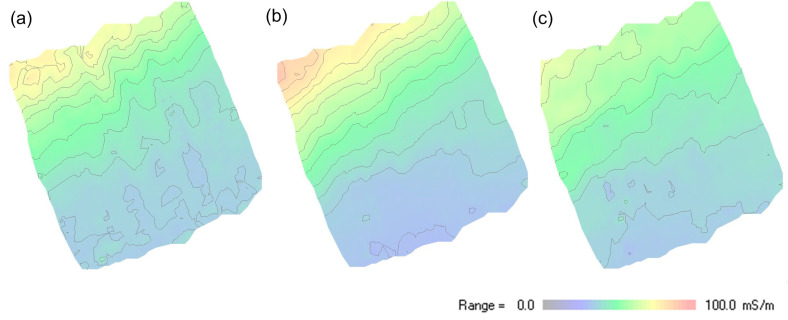
Apparent electrical conductivity (mS/m) of the 25 m × 25 m mapping area shown in Figure 8 above, measured with the MEMIS at different transmitter-receiver coil separations: (**a**) coil separation 90 cm; (**b**) coil separation 170 cm; and (**c**) coil separation 370 cm.

Figure 8 shows the mapping results of data collected in an area of 25 m × 25 m located on the shoreline of the ‘Apetloner Meierhoflacke’, one of the soda lakes located on the eastern side of lake Neusiedlersee in Austria. About half of the investigated region is located outside the lake; the other half is covered with shallow water with a depth of up to 30 cm. The measurement was conducted with the MEMIS with a frequency of 10 kHz and transmitter–receiver coil separations of 90 cm, 170 cm, and 370 cm. Complementary measurements were conducted with the CMD Explorer (manufactured by GF-instruments), which operates at a frequency of approximately 10 kHz and has transmitter–receiver coil separations of 148 cm, 282 cm, and 449 cm. Both systems were hand-carried back and forth across the target area, as shown in Figure 8a, keeping the spatial orientation of the instruments constant during the entire mapping. Measurement results are displayed in Figure 8b (Modular EMI System, coil separation 170 cm) and Figure 8c (CMD Explorer, coil separation 148 cm) in a 2-D plot in false color rendering. Figure 9 presents data obtained at the same location with the MEMIS with different transmitter-receiver coil separations. The data that were used to create Figure 8 and Figure 9 are provides as supplementary material (see section ‘Supplementary Materials’).

Results presented in Figure 7, Figure 8 and Figure 9 demonstrate that the proposed MEMIS can provide data with comparable quality as those measured with a commercial instrument. Hence, we consider that the instrument is ready for further use in teaching and research projects. As presented above, the MEMIS can be adjusted to produce data with high sensitivity close to the surface (as presented in Figure 7) or for deeper investigations (Figure 8), which require the use of two different commercial instruments (i.e., the CMD Mini-Explorer and the CMD Explorer). Hence, the proposed MEMIS clearly provides some advantages, regarding its flexibility to adjust to different geometries to reach different nominal depths of investigation.

## 4. Discussion

An experimental terrain conductivity-meter instrument was designed and built from primarily readily available components and sub-systems. The instrument supports up to four receiver coil antennas for the simultaneous recording of apparent conductivities at four different depths of investigation. The system is modular and offers the possibility to change the separation between the transmitter (Tx) and receivers (Rx) towards the collection of data with variable resolution and depth of investigation. With these properties, the proposed instrument offers an alternative to commercial instruments, which commonly build on a rigid structure and a fixed separation between the transmitter and receiver. In case of GF-instruments, for instance, the collection of data with different resolution and depth of investigation requires the purchase of different instruments (i.e., build with different lengths). Such an alternative might be challenging for research or academia. Moreover, different projects, requiring variable depths of investigation, may also require different instruments. Other devices, such as instruments from Dualem Inc. (Milton, ON, Canada) permit the simultaneous collection of data with horizontal and vertical configurations, yet the separation between the transmitter and receiver is also fixed, resulting in a similar limitation as mentioned before. To overcome this issue, the proposed modular EMI device features the option to mount receiver antennas with a larger separation to the transmitter, which favors deeper investigations, or with a short separation to support shallow investigations with higher resolution. Moreover, the operation frequency can be adjusted between 3 kHz and 50 kHz to enhance the resolution and depth of investigation in the measurements. Here, we want to make it clear that the user needs to select the separation between transmitter and receiver antennas as well as the operation frequency within an adequate range to maintain the conditions for low-induction number and permit a direct computation of $\sigma_{a}$ using the approach proposed by McNeil (see Equation (6)).

Particular features in the design and operation of the MEMIS make this instrument different to commercial instruments like, e.g., the CMD-Explorer from GF-Instruments:

1. The mechanical structure of the MEMIS consists of a pair of rigid but light-weight tubes that are joined together with shorter tubes to a ladder-like structure. The resulting structure provides a flexible basis for mounting the various sub-systems. The basic structure has a length of 200 cm, but can be extended with a second segment to a total length of 400 cm.
2. The MEMIS provides mechanical interfaces to mount the device to mobile platforms.
3. The MEMIS uses rectangular (quadratic) transmitter and receiver antennas. This design was chosen because rectangular antennas proved to be easier to manufacture and better to integrate with the mechanical support structure.
4. The MEMIS does not calculate, display, and record apparent conductivities during a measurement in real-time, but rather records raw signals from the antennas and auxiliary sensors without processing directly to a storage device with a bandwidth of 50 kHz. All data processing, such as the filtering of antenna signals, the calculation of signal attenuations, phase differences, and apparent conductivities, and the evaluation of GPS and IMU data sentences, is performed off-line in the digital domain with a dedicated software program. This approach was adapted to keep the hardware design simple, while implementing more complex processing steps in PC software.

The availability of raw data provides flexibility during data processing and opens the possibility to develop an instrument with simultaneous multi-frequency operation. Such an instrument would create a Tx signal that is composed of a sum of sinusoidal waveforms with different frequencies and feed this signal into the Tx antenna loop. This approach would permit the collection of data with the same separation between Tx and Rx antennas but different frequencies simultaneously. In the same direction, the access to the actual Δφ measurements (instead of transformed $\sigma_{a}$ values) can be fully exploited with adequate modeling and inversion algorithms to resolve for vertical variations in the electrical conductivity. However, the use of different simultaneous frequencies, as well as modeling and inversion are beyond the scope of this manuscript and should be addressed in further studies.

As described in Section 2.3, antenna signals are digitized and recorded with a default sampling rate of 192 kSamples/s. During processing, the phase differences between Tx signal and the Rx signals are calculated using a sequence of Fast Fourier Transforms (FFTs) with a default window size of 19,200 samples, resulting in a data rate of 10 samples/s for calculated conductivity values. This FFT window size can be adapted during data analysis to achieve different data rates for calculated conductivity values, and to influence the signal to noise ration of calculated conductivity values. The selection of a larger FFT window size will decrease the data rate but can be expected to also improve noise performance, which may be of particular interest for the investigation of materials which create only small transmitter–receiver phase shifts (i.e., have low conductivity).

A certain drawback of recording raw data is a high data storage requirement that amounts to 300 MByte for one minute of recorded raw data. However, high-capacity SD memory cards are available and can store the data accumulated during a whole working day.

The MEMIS integrates a GPS unit and an orientation sensor. This feature facilitates the operation of the instrument, since the operator does not have to hand-carry a separate GPS antenna pole. Data from the position and orientation sensors allow for the 3D position and orientation of the instrument. Such information is required during the analysis of the readings and for the construction of maps presenting the variations of the $\sigma_{a}$.

The MEMIS is accompanied by an interactive software package that implements many functions that are required to manage and process the recorded raw data and create .csv files as an output. These files can be read, processed, and displayed by standard software packages such as MS-Excel (v2019) or QGIS (v3.24), or custom software programs implemented in, e.g., Python (v3). Alternatively, the Modular EMI System software v1.0 itself is equipped with functions to read and graphically display the contents of .csv output files created by it. However, further work is required to build user-friendly interfaces for the configuration of the instrument as well as the processing of the data.

Conductivity data obtained in field tests of the instrument consistently show a good match with data obtained with the CMD Explorer, which we use as a reference instrument. Tests further revealed that the zero-baseline of the Rx-Tx phase difference measurements and derived conductivity calculations is showing a drift that starts right after the power-up of the system and reaches an equilibrium after approximately 10 min. A zero-baseline drift is also observed when the room temperature of the laboratory is changed. For practical work with the MEMIS instrument, we thus recommend powering up the recorder 10 min before the first measurement to allow for temperature equilibration, and to perform regular calibrations to compensate for temperature changes of the test site environment. The electronic assembly of the MEMIS is currently not protected against dust or spray water, so care must be taken to avoid exposure to rain or to a dusty environment.

Temperature drifts of EMI instruments are routinely reported in studies, together with strategies to deal with those drifts [5,20,41,42]. Typical mitigation strategies for temperature-induced drifts include: (1) allowing the system to be in equilibrium with the ambient conditions at the beginning of a measurement [20]; (2) nulling of the instrument at the beginning of each day and then several times per day [41]; (3) avoid direct sunlight during a survey, since a change from direct sunlight to shadow (and back) can produce a significant drift [20]; and (4) perform a measurement at the beginning and at the end of a survey on exactly the same position, then calculate the difference between these two measurements and correct the entire measurement for the observed drift using (linear) interpolation [20]. An alternative model-based drift correction method that measures electrical system parameters of Tx and Rx antennas and employs these data to correct for drifts during post-processing was developed and tested by Forschungszentrum Jülich GmbH, Germany [22,23]. This method was also used to select optimal measurement frequencies based on an ambient noise level analysis.

The zero baseline of the MEMIS is currently determined by comparing measurement results obtained with this instrument with results obtained with a commercial CMD Explorer and adjusting the zero baseline accordingly. Alternative approaches to determine the zero baseline have been tested, in particular: (1) Positioning the instrument at an approximately 5 m height above ground on a permanent wooden observation platform that is located next to our field test site. (2) Positioning the instrument at an approximately 3 m height above ground on a temporary portable light-weight wooden rack that was erected next to our field test site. (3) Manually orienting and holding the instrument with its long axis vertically aligned. The systematic evaluation of these zero baseline methods is still in progress.

The growing number of applications have made the mapping with LIN-EMI instruments a powerful technique for mapping extensive areas as required for environmental and hydrogeological studies, in particular for the emerging discipline of agrogeophysics [43]. However, inversion of the data is needed to resolve for the variations in the electrical conductivity at depth. The inversion of data collected with commercial instruments is usually limited by the small number of measurements at a given position (for instance three datasets using the CDM-Explorer or CMD-Mini-Explorer). In contrast, the proposed MEMIS offers the possibility to collect data with varying depth sensitivity at a given point by changing the geometry (transmitter–receiver separations) and acquisition frequencies. Increasing the number of measurements per point allows reduction of the uncertainties during the inversion of conductivity models [44,45]. Moreover, the availability of the raw data before transformation to $\sigma_{a}$ using the LIN approach could be further exploited to improve the quantification of electrical conductivity as well as the magnetic susceptibility using, for instance, stochastic approaches [46,47]); thus, permitting an improved subsurface investigation.

## 5. Conclusions

We developed, built, and tested an experimental EMI instrument that consists to a large extent components and sub-systems that are commercially available off-the-shelf items. Its mechanical and electronic design, and the data processing approach differ significantly from the one of commercial instruments. Measurement results obtained with this instrument compare well with results obtained with a commercial CMD Explorer which we used as reference instrument. Field tests of the instrument demonstrated that the device is easy to handle by a single operator, and its build is sufficiently rugged for practical field operations. We consider the instrument suitable for further use in teaching and research projects, where we expect that it will provide advantages regarding its flexibility to adjust to different geometries to reach different nominal depths of investigation.

## Figures and Tables

**Table 1:** MEMIS v2, long configuration: design details of the transmitter coil antenna (Tx), the monitor antenna (Tx-Mon), and three receiver coil antennas (Rx-2 to Rx-4).

Description	Unit	Tx	Tx-Mon	Rx-1	Rx-2	Rx-3	Rx-4
Coil diameter	cm	27.5	27.5	-	27.5	27.5	29.5
Wire diameter	mm	0.75	0.9	-	0.5	0.5	0.5
Turns	1	20	1	-	6	20	20
Resistance R	Ohm	0.70	0.02	-	0.48	1.58	1.70
Inductance L	μH	400	1	-	40	436	473
Tx-Rx coil separation s	cm	-	-	-	90	170	370
Depth of exploration, HCP	m	-	-	-	1.44	2.72	5.90
Depth of exploration, VCP	m	-	-	-	0.68	1.29	2.81

## Data Availability

The original data presented in the study are openly available in DOI: https://doi.org/10.48436/kssdd-0cz48 (accessed on 16 May 2024).

## Supplementary Materials

The following supporting information can be downloaded at: https://www.mdpi.com/article/10.3390/s24134159/s1, The .zip file contains 7 files with data tables that were used to create Figure 7, Figure 8 and Figure 9. Data were obtained with the MEMIS system. In addition, one .pdf is provided that explains the column headers used in the data table files.
