# Supplementary material for: Design, Development and Application of a Modular Electromagnetic Induction (EMI) Sensor for Near-Surface Geophysical Surveys
Source: Sensors (Basel). 2024 Jun 26;24(13):4159. doi: 10.3390/s24134159 (PMC11244430; doi:10.3390/s24134159)
Supplement: Supplementary file 1 [file sensors-24-04159-s001.zip › MEMIS - Data/MEMIS - Description of Column Headers.pdf]

Label		*Description*
File		Raw data file name
Nr		Number of segment in raw data file name
Valid		Valid = 1 … Data sample is valid; Valid = 0 … Data sample is skipped.
Time		Time of data sample in seconds
NanoSec		Time offset in nanoseconds
Latitude		Latitude
Longitude		Longitude
Elevation		Elevation
Local_X		Coordinates in local coordinate system, origin is position of first data sample
Local_Y		Coordinates in local coordinate system
Dist		Distance from origin of local coordinate system
Frequency		Frequency of Tx signal
Ampl_Tx		Amplitude of Tx-Mon signal
Phase_Tx		Phase of Tx-Mon signal, in degrees
PhStep_Tx		Difference of phase between two consecutive samples of Tx-Mon signal
Ampl_1		Amplitude of Rx-1 signal
Phase_1		Phase of Rx-1 signal, in degrees
PhStep_1		Difference in phase between two consecutive Rx-1 signals, in degrees
Ampl_2		Amplitude of Rx-2 signal
Phase_2		Phase of Rx-2 signal, in degrees
PhStep_2		Difference in phase between two consecutive Rx-2 signals, in degrees
Ampl_3		Amplitude of Rx-3 signal
Phase_3		Phase of Rx-3 signal, in degrees
PhStep_3		Difference in phase between two consecutive Rx-3 signals, in degrees
Ampl_4		Amplitude of Rx-4 signal
Phase_4		Phase of Rx-4 signal, in degrees
PhStep_4		Difference in phase between two consecutive Rx-4 signals, in degrees
dPhaseR_1		Phase of Rx-1 signal, relative to Tx-Mon signal, in degrees
dPhaseR_2		Phase of Rx-2 signal, relative to Tx-Mon signal, in degrees
dPhaseR_3		Phase of Rx-3 signal, relative to Tx-Mon signal, in degrees
dPhaseR_4		Phase of Rx-4 signal, relative to Tx-Mon signal, in degrees
dPhaseC_1		Phase of Rx-1 signal, relative to Tx-Mon signal, corrected with calibration value, in degrees
dPhaseC_2		Phase of Rx-2 signal, relative to Tx-Mon signal, corrected with calibration value, in degrees
dPhaseC_3		Phase of Rx-3 signal, relative to Tx-Mon signal, corrected with calibration value, in degrees
dPhaseC_4		Phase of Rx-4 signal, relative to Tx-Mon signal, corrected with calibration value, in degrees
Cond_1		Apparent conductivity of Rx-1 signal
Cond_2		Apparent conductivity of Rx-2 signal
Cond_3		Apparent conductivity of Rx-3 signal
Cond_4		Apparent conductivity of Rx-4 signal
